# Assessment of Malaria Burden and Community Response to Control and Management Programs

**DOI:** 10.7759/cureus.85624

**Published:** 2025-06-09

**Authors:** Fahmida Khatoon, Naglaa M Shalaby, Aida El Alginawi, Zeinab Alaagib Ahmed Kara, Maha M Abdul-Latif, Gamal Eldin Mohamed O Elhussein, Abdullah Ahmed Alreshidi, Fayez S Alreshidi, Fatima H Alnourain, Manal Elzein Musa Ismail

**Affiliations:** 1 Department of Biochemistry, College of Medicine, University of Ha’il, Ha’il, SAU; 2 Department of Medical Parasitology, Faculty of Medicine, Mansoura University, Mansoura, EGY; 3 Department of Microbiology, Faculty of Medicine, Northern Border University, Arar, SAU; 4 Department of Biochemistry, Faculty of Medicine, Najran University, Narjan, SAU; 5 Department of Ophthalmology, Faculty of Medicine, Northern Border University, Arar, SAU; 6 Department of Pediatrics, College of Medicine, University of Ha’il, Ha’il, SAU; 7 Department of Medicine, Vision College, Riyadh, SAU; 8 Department of Family and Community Medicine, College of Medicine, University of Ha’il, Ha’il, SAU; 9 Department of Family and Community Medicine, Faculty of Medicine, Najran University, Najran, SAU; 10 Department of Obstetrics and Gynecological Nursing, Faculty of Applied Medical Sciences, Buraydah College, Buraydah, SAU

**Keywords:** malaria, medical managment, outcomes, patient satisfaction, public health and safety

## Abstract

Introduction: Malaria continues to be a significant public health issue, particularly in tropical and subtropical regions. Despite extensive efforts to reduce its burden through various control programs, malaria remains a major cause of morbidity and mortality. This study aims to assess the burden of malaria in a community and evaluate the community's response to malaria control and management programs.

Objective: This study aimed to assess the prevalence of malaria and evaluate the community's awareness, participation, and response to malaria control and management programs.

Methodology: This cross-sectional study included 550 participants from a community-based population. Data were collected through interviews, surveys, and medical records to determine malaria prevalence, treatment-seeking behavior, and knowledge of malaria control measures such as insecticide-treated nets (ITNs), indoor residual spraying (IRS), and antimalarial treatments.

Results: The study found a malaria prevalence of 32% (176 participants), with higher rates in females at 35% (96 out of 275) compared to males at 30% (81 out of 275). A total of 85% (468 participants) recognized malaria symptoms. Awareness of prevention methods was reported by 72% (396 participants) for ITNs and 58% (319 participants) for IRS. Regarding treatment-seeking behavior, 60% (330 participants) visited health facilities, while 25% (138 participants) relied on traditional healers. Participation in malaria control programs was moderate: 62% (341 participants) reported using ITNs, and 50% (275 participants) had participated in IRS activities. Identified barriers included a lack of awareness in 30% (165 participants) and high costs in 20% (110 participants). Socioeconomic status influenced program engagement, with higher participation observed among individuals from wealthier households.

Conclusion: Although malaria control and management programs have had a positive impact, only 62% (341 participants) used ITNs and 50% (275 participants) engaged in IRS, indicating a gap in community adherence. Despite 85% (468 participants) being aware of malaria symptoms, knowledge and practical application of preventive measures remain suboptimal. Improving public education, enhancing access to malaria control tools, and addressing socioeconomic barriers are essential to effectively reduce the malaria burden in the community.

## Introduction

Malaria poses one of the most critical public health threats worldwide, while it primarily affects sub-Saharan Africa and Southeast Asia, together with Latin American territories [1]. The pathogen causing the illness originates from *Plasmodium *parasites that spread from contaminated *Anopheles *mosquitoes to humans via mosquito stings. Two decades of malaria control progress have not eliminated the disease, which remains one of the main health threats that particularly affects developing nations with fragile healthcare networks [2]. The World Health Organization reported a total of 229 million malaria cases worldwide during 2019, which resulted in 409,000 fatalities, with most fatalities occurring among children under age five in Africa. Malaria burden creates two major health challenges that additionally act as developmental obstacles by negatively affecting worker productivity and medical expenses in addition to social welfare throughout several regions [3].

The fight against malaria includes preventive and therapeutic actions, which include insecticide-treated nets (ITNs) distribution as well as indoor residual spraying (IRS) and immediate availability of antimalarial medications [4]. Two affordable treatment methods called artemisinin-based combination therapies (ACTs) have helped lower mortality rates throughout areas where they have achieved broad distribution [5]. The implementation of mass distribution campaigns that include ITN distribution and IRS program implementations has shown success in lowering malaria transmission rates across different areas. Despite achievements in eliminating malaria, there are multiple regions where transmission remains endemic because of resistance against insecticides, together with the appearance of drug-resistant strains and ecological changes [6, 7]. Gaps in public response to malaria prevention programs exist because of three main obstacles, which include logistics issues, cultural beliefs, and socioeconomic factors. Slender healthcare infrastructure combined with insufficient healthcare access persists as major obstacles for the diagnosis and treatment of malaria in rural and remote locations [8]. Affected communities have substantial deficiencies in understanding how to prevent malaria and the need for early diagnosis, together with proper treatment adherence. Research findings indicate a wide awareness of malaria symptoms exists among the population, yet population understanding of proven prevention methods, including frequent ITN usage and IRS application, remains insufficient [9]. Whether a community’s actions are positive or negative decides the success of malaria control efforts. Even though people usually understand malaria symptoms, they seldom follow practical ideas like regularly using ITNs, collaborating in IRS, and sticking to antimalarial treatment [8]. In some places, people keep using home remedies and local healers because old beliefs influence their choices for seeking treatment [9]. Besides, access to care, education, and socioeconomic conditions in a community play important roles in controlling diseases [10].

The study investigates both the malaria disease impact alongside community reactions to malaria prevention programs in endemic areas. The research focuses on assessing malaria incidence alongside community behavior related to treatment selection and malaria control activities to determine knowledge gaps and implementation obstacles, and factors regarding community commitments. 

The primary objective of the study is to assess the malaria burden and evaluate the community’s response to malaria control and management programs.

## Materials and methods

This retrospective, community-based cross-sectional study was conducted from January to July 2024 in a malaria-endemic area, with research oversight from the University of Ha'il, Ha'il, Kingdom of Saudi Arabia. A total of 550 participants were included to assess the malaria burden and evaluate community response to control and management programs.

The sample size was determined using the standard formula for cross-sectional studies:

$n = \frac{Z^2 \cdot p (1 - p)}{d^2}$

where: Z = 1.96 (standard normal deviate for a 95% confidence interval); p = 0.5 (assumed prevalence of malaria, to ensure maximum sample size); d = 0.05 (desired margin of error)

The calculated sample size was 384. However, to increase the power and account for potential data loss or non-response, the final sample included 550 participants.

Participants included in the study were those of any age residing in the study area who had been diagnosed with malaria within the study period based on clinical symptoms and confirmation through rapid diagnostic tests (RDTs) or microscopy. Only individuals who were willing to participate and provided informed consent were included. Participants were excluded if they had a confirmed diagnosis of malaria but declined to participate, had incomplete medical records or missing essential data such as age, malaria status, or survey responses, or were unable to effectively communicate due to illness, cognitive limitations, or language barriers.

Data collection

Data for this study were collected through surveys conducted with 550 individuals from the community. The data collected included demographic information such as age, gender, socioeconomic status, and education level (Appendix A). It underwent content validation by a panel of public health experts at the University of Ha'il. Face validity was established to ensure clarity, cultural relevance, and simplicity of language. Construct validity was evaluated during a pilot study involving 30 participants, approximately 10% of the final sample size, to assess reliability and internal consistency. Modifications were made based on feedback, and the tool achieved a Cronbach’s alpha score of 0.78, indicating strong internal consistency. Data on malaria prevalence were collected through self-reports of malaria cases, confirmed by medical records or rapid diagnostic tests. Additionally, participants were asked about their knowledge of malaria symptoms, prevention measures (such as ITNs and IRS), and treatment options. Treatment-seeking behavior was also assessed by inquiring where individuals typically sought care for malaria, including health facilities, traditional healers, or home remedies. Finally, the community's participation in malaria control programs, including the use of ITNs, attendance at health education sessions, and involvement in IRS campaigns, was evaluated (Appendix A). The data collected were then analyzed to assess the relationship between community response and malaria control efforts. To ensure accuracy, self-reported malaria cases were cross-verified with medical records and diagnostic confirmations via RDTs or microscopy.

Statistical analysis

Data were analyzed using IBM SPSS Statistics software, version 26 (IBM Corp., Armonk, NY). Chi-square tests were applied to determine associations between demographic factors (e.g., age, education, and income) and knowledge of malaria control measures. Independent samples t-tests were used for comparing mean differences between binary groups (e.g., male vs. female awareness scores). A p-value of <0.05 was considered statistically significant. 

## Results

Data were collected from 550 participants with an average age of 35.4 ± 12.8 years. The sample included 300 (54.5%) males and 250 (45.5%) females. Regarding education level, 220 (40.0%) participants had only primary education, 193 (35.1%) had secondary education, and 137 (24.9%) had higher education. Socioeconomic status was categorized as follows: 248 (45.1%) were in the low-income group, 220 (40.0%) in the middle-income group, and 82 (14.9%) in the high-income group (Table 1).

**Table 1:** Demographic characteristics of the participants

Characteristic	Total (n = 550)	Male (n = 300)	Female (n = 250)	p-value
Mean age (years)	35.4 ± 12.8	36.2 ± 12.3	34.0 ± 13.2	0.11
Education level				0.05
- Primary education	220 (40.0%)	114 (38.0%)	106 (42.4%)	
- Secondary education	193 (35.1%)	111 (37.0%)	82 (32.8%)	
- Higher education	137 (24.9%)	75 (25.0%)	62 (24.8%)	
Socioeconomic status				0.02
- Low	248 (45.1%)	129 (43.0%)	119 (47.6%)	
- Middle	220 (40.0%)	126 (42.0%)	94 (37.6%)	
- High	82 (14.9%)	45 (15.0%)	35 (14.0%)	

Among the 550 participants, 176 (32.0%) were diagnosed with malaria, with a slightly higher prevalence among females (34.4%) than males (30.0%), though the difference was not statistically significant (p = 0.09). Regarding treatment-seeking behavior, a majority (60.0%) sought care from health facilities, with males (62.0%) more likely than females (57.6%) to do so. Notably, reliance on traditional healers was higher among females (28.8%) compared to males (22.0%) (Table 2).

**Table 2:** Malaria prevalence and treatment-seeking behavior in the study group

Variable	Total (n = 550)	Male (n = 300)	Female (n = 250)	p-value	Test statistic
Malaria cases diagnosed	176 (32.0%)	90 (30.0%)	86 (34.4%)	0.09	χ² = 2.87
Treatment-seeking behavior				0.01	χ² = 9.21
- Health facility	330 (60.0%)	186 (62.0%)	144 (57.6%)		
- Traditional healers	138 (25.1%)	66 (22.0%)	72 (28.8%)		
- Home remedies	82 (14.9%)	48 (16.0%)	34 (13.6%)		

A total of 468 (85.1%) participants correctly identified malaria symptoms. Awareness of prevention methods was lower: 396 (72.0%) participants were aware of ITNs, 319 (58.0%) knew about IRS, and 358 (65.1%) understood the importance of antimalarial drugs. Awareness of ITNs was higher among females, with 206 (74.9%) out of 275 females reporting knowledge, compared to 190 (69.1%) out of 275 males. However, knowledge about IRS and antimalarial drugs was similar across genders (Table 3).

**Table 3:** Knowledge of malaria symptoms and prevention ITNs: insecticide-treated nets; IRS: indoor residual spraying

Knowledge of malaria	Total (n=550)	Male (n=300)	Female (n=250)	p-value	Chi-square (χ²)
Correctly identified symptoms	468 (85.1%)	249 (83.0%)	220 (88.0%)	0.04	4.17
Awareness of ITNs	396 (72.0%)	210 (70.0%)	186 (74.4%)	0.06	3.54
Awareness of IRS	319 (58.0%)	165 (55.0%)	155 (62.0%)	0.12	2.42
Knowledge of antimalarial drugs	358 (65.1%)	189 (63.0%)	170 (68.0%)	0.08	3.01

Regular use of ITNs was reported by 341 (62.0%) participants, including 192 (64.0%) out of 300 males and 149 (60.0%) out of 250 females. Participation in IRS campaigns was reported by 275 (50.0%) participants, with 150 (50.0%) males and 125 (50.0%) females, showing no significant gender differences. Attendance at health education sessions was noted in 220 (40.0%) participants, with slightly higher attendance among females (105 (42.0%)) compared to males (114 (38.0%)) (Table 4).

**Table 4:** Community participation in malaria control programs ITNs: insecticide-treated nets; IRS: indoor residual spraying

Participation in programs	Total (n=550)	Male (n=300)	Female (n=250)	p-value	Chi-square (χ²)
Use of ITNs regularly	341 (62.0%)	192 (64.0%)	149 (59.6%)	0.1	2.67
Participation in IRS campaigns	275 (50.0%)	144 (48.0%)	131 (52.4%)	0.15	2.07
Attendance at health education sessions	220 (40.0%)	126 (42.0%)	95 (38.0%)	0.14	2.18

Lack of awareness was reported as the most common barrier by 165 (30.0%) participants, followed by inaccessibility of resources by 138 (25.1%) and high cost of prevention tools such as ITNs by 110 (20.0%). Cultural barriers, including reliance on traditional healers, were noted by 83 (15.1%) participants, while 55 (10.0%) reported that the distance to healthcare facilities was a major barrier to seeking care (Table 5).

**Table 5:** Barriers to malaria prevention and control participation

Barrier	Total (n=550)	Male (n=300)	Female (n=250)	p-value	Chi-square (χ²)
Lack of awareness	165 (30.0%)	84 (28.0%)	80 (32.0%)	0.07	3.27
Inaccessibility of resources	138 (25.1%)	78 (26.0%)	60 (24.0%)	0.1	2.68
Cost of prevention tools	110 (20.0%)	57 (19.0%)	53 (21.2%)	0.08	3.01
Cultural barriers (traditional healers)	83 (15.1%)	42 (14.0%)	40 (16.0%)	0.06	3.58
Distance to health facilities	55 (10.0%)	36 (12.0%)	20 (8.0%)	0.09	2.91

## Discussion

The research results help understand both malaria’s impact on the community along with people’s responses to control and management initiatives. The implementation of control programs has not eliminated the major public health impact of malaria, especially in targeted territories. Among the 550 participants studied, this research revealed substantial malaria cases because 32% experienced recent malaria attacks. A high percentage of 85% of members within the community properly identified malaria symptoms despite the significant burden of malaria affecting them. Insufficient understanding existed among the community members about all available preventive actions against malaria. The research showed that ITNs received attention from 72% of people, but regular use reached only 62%, and IRS campaigns enrolled 50% of the population. The different rates at which people participate in malaria control programs demonstrate multiple elements that affect how well these interventions are adopted by the population [11]. Socioeconomic aspects seemed to influence why most participants knew about ITNs and IRS, but showed reduced practice of these interventions. Previous research confirms that poverty, together with insufficient healthcare infrastructure, acts as a primary roadblock against effective malaria prevention in various areas [12].

Participants displayed different behaviors when seeking treatment, according to the study findings. A total of 60% of people visited health facilities, but traditional healers treated 25% of patients, while home remedies received 15% of cases. The findings demonstrate how rural populations, together with socioeconomically disadvantaged regions, maintain their use of unlicensed healthcare providers [13]. The integration of traditional healers into malaria control programs through proper training about immediate treatment practices will strengthen malaria management across these communities [14]. Knowledge about malaria prevention programs both inside the household (IRS) and outside the household (average-risk chemoprophylaxis with antimalarial drugs) remained limited, even though people showed good awareness of malaria itself. Less than half of the participants mentioned using antimalarial drugs to prevent malaria, while 65% demonstrated familiarity with the drugs [15]. 

People failed to join malaria control programs primarily because they lacked awareness about prevention approaches by 30%, along with limited access to resources at 25%. Twenty percent of participants stated that high costs prevented them from purchasing malaria prevention equipment, including ITNs, together with healthcare services. The findings support the need for malaria control initiatives to address both economic limitations and resource distribution when developing their programs. The finding supports existing research, which demonstrates that educational efforts contribute significantly to improving health conduct and strengthening public health intervention success [16]. The implementation of malaria control programs depends heavily on the involvement of community members [17]. The research showed that health education participation as well as malaria prevention program attendance were moderately active, but additional improvements could be made [18]. 

Future investigations about how traditional healer integration into malaria control initiatives performs in regions with restricted formal healthcare may provide important knowledge. Widespread participation alongside consistent use of preventive tools remains a challenge despite existing progress in malaria awareness elevation and control implementation. The community needs effective solutions to break down socioeconomic barriers and improve resource accessibility while strengthening community support in order to decrease malaria rates. This study has several limitations that should be considered when interpreting the findings. Being cross-sectional in design, it limits the ability to establish causality between observed factors and malaria-related outcomes. The reliance on self-reported data introduces the potential for recall and social desirability biases, particularly regarding malaria episodes, ITN use, and treatment-seeking behavior. Selection bias may also be present, as individuals who were not at home during data collection were excluded, possibly affecting the representativeness of the sample. 

## Conclusions

This study highlights the continuing burden of malaria and the moderate response of the community to malaria control and management programs. While awareness of malaria exists, there are clear gaps in knowledge about effective prevention methods. Efforts to improve malaria control should include enhanced community education, increased access to prevention tools, and strategies to address barriers to participation in control programs. A stronger community response will be key to reducing malaria incidence and improving public health outcomes.

## Appendices

Appendix A

Malaria Community Survey Questionnaire

Study Title: Community Response to Malaria Control and Management Programs Location: [Insert Name of Community] Date: [] Participant ID: []

Age: ______ years

Gender: ☐ Male  ☐ Female

Education Level: ☐ No formal education ☐ Primary ☐ Secondary ☐ Higher

Occupation: ___________________

Household monthly income: ☐ <1000 SAR ☐ 1000-3000 SAR ☐ 3001-5000 SAR ☐ >5000 SAR

Number of people in the household: ______

Have you had malaria in the past 12 months? ☐ Yes  ☐ No

If yes, how was the diagnosis confirmed? ☐ Rapid Diagnostic Test (RDT) ☐ Microscopy ☐ Symptoms only ☐ Don’t know

How many times have you had malaria in the last year? _______

Can you identify common symptoms of malaria? (Select all that apply) ☐ Fever ☐ Chills ☐ Sweating ☐ Vomiting ☐ Headache ☐ Body aches

Are you aware of the following malaria prevention methods? a. Insecticide-Treated Nets (ITNs): ☐ Yes ☐ No b. Indoor Residual Spraying (IRS): ☐ Yes ☐ No c. Antimalarial drugs: ☐ Yes ☐ No

Have you ever received any information or education about malaria? ☐ Yes  ☐ No

Section D: Practices and Participation

Do you sleep under an insecticide-treated net regularly? ☐ Yes  ☐ No

Has your household been sprayed with insecticide in the past year? ☐ Yes  ☐ No

Have you attended any health education sessions on malaria? ☐ Yes  ☐ No

Do you take antimalarial drugs as a preventive measure? ☐ Yes  ☐ No

Where do you usually seek treatment when you or a family member has malaria? ☐ Government hospital/clinic ☐ Private clinic ☐ Traditional healer ☐ Home remedies

How long after symptoms begin do you usually seek care? ☐ Same day ☐ 1-2 days ☐ After 3 or more days ☐ Don’t seek care

Do you trust traditional healers more than medical facilities? ☐ Yes  ☐ No

What prevents you from using malaria prevention or treatment methods? (Select all that apply) ☐ Lack of awareness ☐ High cost ☐ Limited access to health services ☐ Cultural beliefs ☐ Distance to facility

Do you believe malaria can be prevented? ☐ Yes  ☐ No

Do you think current malaria control programs are effective in your community? ☐ Yes  ☐ No ☐ Not sure

Consent

☐ I have been informed about the purpose of this survey and I voluntarily agree to participate.

Signature/Thumbprint: ___________________  Date: _______________
